# Fermentative Characteristics and Metabolic Profiles of Japanese Apricot Juice Fermented with *Lactobacillus acidophilus* and *Torulaspora delbrueckii*

**DOI:** 10.3390/foods13213455

**Published:** 2024-10-29

**Authors:** Benjawan Papun, Pairote Wongputtisin, Apinun Kanpiengjai, Tippapha Pisithkul, Phayungsak Manochai, Kamonwan Manowan, Anong Atsaneechantra, Ni-orn Chomsri

**Affiliations:** 1 Agricultural Technology Research Institute, Rajamangala University of Technology Lanna, Lampang 52000, Thailandpkmanochai1972@gmail.com (P.M.);; 2 Program in Biotechnology, Faculty of Science, Maejo University, Sansai, Chiang Mai 50290, Thailand; pairotewong@gmail.com (P.W.); tpisithkul@gmail.com (T.P.); 3 Division of Biochemistry and Biochemical Innovation, Department of Chemistry, Faculty of Science, Chiang Mai University, Chiang Mai 50200, Thailand; apinun.k@cmu.ac.th

**Keywords:** Japanese apricot, *Prunus mume*, fermentation, *Lactobacillus*, *Torulaspora*

## Abstract

Functional fermented fruit juices produced using a combination of non-*Saccharomyces* yeast and lactic acid bacteria (LAB) are relatively unexplored. The effects of three inoculation protocols, single inoculation with *Lactobacillus acidophilus* (*La*), single inoculation with *Torulaspora delbrueckii* (*Td*), and co-culture of both *La* + *Td*, on the physicochemical, microbiological, sensory properties, and metabolic profile of fermented JA juices after 24 h at 30 °C were investigated. Uninoculated (UI) Japanese apricot (JA) juice was used as a control. The results show significant increases in the color intensity of the fermented-JA juices, whereas an enhancement of total phenolic contents is observed in the fermented JA-juices acquired through the use of *La* for both single and co-culture inoculations. The colony counts of LAB and yeast in the inoculated JA juices increased by approximately 2.0 and 1.7 log CFU/mL at 24 h, respectively. The antibacterial activity of JA juices against four pathogenic bacteria was detected. All JA juices exhibited antimicrobial activity against the tested pathogenic strains, with strong antibacterial properties of *La*-fermented juice being recorded against *Bacillus cereus* at the lowest MIC of 124 µL/mL. Additionally, *La* + *Td*-fermented and UI-JA juices demonstrated comparable anticancer activity against HT-29 cells with IC50 values of 823.37 and 754.87 µg/mL, respectively. Furthermore, a total of 995 compounds was identified as differential fermentation metabolites through non-targeted metabolome analysis across different fermentation groups. These findings illustrate the significant potential of using JA juice for *La* and *Td* fermentation to develop functional juices.

## 1. Introduction

Nowadays, fermented beverages, like fruit wine, yogurt, kombucha, kefir, and fruit juice, have gained global popularity due to their potential health benefits as functional foods [1,2]. These drinks are known for enhancing digestion, boosting immunity, and providing probiotics, which may help protect against chronic and metabolic diseases [3,4]. Fermentation also improves nutrient availability and creates distinct flavors, which appeal to health-conscious consumers [5]. As interest in health-focused diets rises, fermented beverages are increasingly recognized in the food industry for their benefits beyond basic nutrition. Moreover, a greater emphasis on healthier living and rising health care concerns are driving the shift toward healthier dietary choices [6]. However, despite consumer beliefs in the additional health benefits of fermented beverages, the industry continues to face challenges in developing products that meet evolving consumer preferences.

*Prunus mume*, commonly known as Japanese apricot (JA), *ume* in Japan, and *mei* in China, has been cultivated across East Asia for centuries for its culinary, medicinal, and ornamental uses [7,8,9]. JA fruit is abundant in volatile compounds and serves as a rich source of essential nutrients, including organic acids, amino acids, minerals, edible fiber, and phenolic compounds [10,11,12]. The versatile JA fruit can be processed into various products, such as sauces, juices, pickles, garnishes, and alcoholic beverages, like wine and liqueurs [13,14,15]. Due to its notable nutritional, pharmacological, and biological properties, numerous studies have explored its potential in food processing, including in products that involve fermentation. For instance, JA fruit has been used in two-stage fermentation with *Saccharomyces* (*S.*) *cerevisiae* and *Acetobacter pasteurianus*, resulting in vinegar with desirable sensory qualities [16]. Another study demonstrated that JA vinegar, with its antioxidative properties, contributed to improved fatigue recovery in exhausted rats [17]. Additionally, the natural fermentation of JA for syrup production resulted in a syrup with favorable functional properties [18]. JA has also been extensively utilized for winemaking; JA wine exhibits a greater antioxidant capacity and contains higher levels of esters and phenolic compounds than JA juice [19]. Moreover, JA co-culture fermentation with *S. cerevisiae* and *Torulaspora* (*T.*) *delbrueckii* enhanced the content of acetate esters, ketones, and terpenes [20]. Additionally, JA wine treated with 50% alcohol had higher concentrations of terpenes and acid–lipid compounds, as well as a more intense aroma when compared with low-alcohol treatments [21].

The research on fermenting JA using LAB is limited. One study found that fermenting 20% JA extract with 15% fructose using *Lactiplantibacillus* (*Lpb.*) *plantarum* for 72–96 h produced a lactic acid juice with optimal sensory properties [22]. A more recent study using a co-culture of *Lpb. plantarum* and *Lacticaseibacillus* (*Lbs.*) *casei* for JA fermentation determined that the fermented JA juice could be used as a natural therapeutic agent for colitis and intestinal inflammation [17]. Despite these documented health benefits, JA remains underutilized. Expanding its application in fermented, bioactive-rich beverages presents a significant opportunity to capitalize on the growing interest in the functional beverage market.

Current research suggests that utilizing yeast or bacteria is part of a promising approach for fermenting JA juice with the goal of producing a fermented beverage. However, most studies have primarily focused on JA wine and vinegar fermentation, with limited attention being paid to the dynamic interactions between different microbial species. Additionally, the challenges associated with using LAB and non-*Saccharomyces* yeast in JA juice fermentation, including co-culture strategies, have not been adequately explored. While *Lactobacillus acidophilus* has been documented to potentially enhance both the functional and sensory properties of fermented beverages [3,23], it is understood that *T. delbrueckii* contributes a degree of complexity to this process through its metabolites [24,25]. However, there are limited studies on the application of these strains in JA juice fermentation. Moreover, the impact of combining these two strains as a co-culture on the quality of fermented JA juice has not been thoroughly investigated. Exploring the use of *Lab. acidophilus* and *T. delbrueckii* in these contexts could highlight their versatility and potential for innovation and diversity in the food and beverage industries. Therefore, this study aimed to investigate the microbiological and physicochemical properties of different fermented JA juices produced using *Lab. acidophilus* and *T. delbrueckii*. An untargeted metabolomic analysis was also conducted to reveal the differences in metabolites of fermented JA juices that were influenced by different inoculation protocols and to provide guidance for the development of product quality. This work provides a practical reference for the development of functional fermented JA juice production through *Lab. acidophilus* and *T. delbrueckii* fermentation.

## 2. Materials and Methods

### 2.1. Raw Materials and Preparation

The Japanese apricot (*Prunus mume*) used in this research was obtained from a plantation located in Fang District, Chiang Mai Province, Thailand. The JA fruits were harvested at the mature-green stage and then ripened at a temperature of 30 ± 2 °C. The selection process involved identifying JA fruits that were uniformly yellow, indicating full ripeness. The fruits were then washed, and only the flesh was separated using a fruit pulper and finisher (RMUTL Model, Lampang, Thailand). The JA pulp was mixed with water at a ratio of 1:7. The mixture was adjusted to achieve the desired total soluble solid content of 13 °C Brix using commercial sucrose. Then, the juice was filtered with an 80-mesh nylon cloth and pasteurized at 90–95 °C for 15 min. Lastly, the juice was cooled down to 30 °C.

### 2.2. Microorganisms and Culture Conditions

*The bacterial strain, Lab. acidophilus* TISTR 1338 (*La*), used in this research was obtained from the culture collection of the Thailand Institute of Scientific and Technological Research (TISTR), while the yeast strain, *T. delbrueckii KE36* (*Td*), was obtained from the Agricultural Technology Research Institute, Thailand. The 2 strains were maintained as stock cultures in 40% (*v*/*v*) glycerol and stored at −80 °C until further use. *Lactobacillus acidophilus* TISTR 1338 was reactivated and propagated for 24 h at 30 °C in de Man, Rogosa, and Sharpe (MRS) broth (HiMedia, Nashik, India). The yeast was reactivated and propagated for 24 h at 30 °C in YEPD broth. The YEPD broth contained (per liter of deionized water) 10 g of yeast extract (Merck, Darmstadt, Germany), 20 g of peptone (Merck, Darmstadt, Germany), 20 g of dextrose (HiMedia, Nashik, India), and 15 g of agar (HiMedia, Nashik, India).

### 2.3. Preparation of Inocula and JA Juice Fermentation

Yeast grown in YEPD broth and the LAB grown in MRS broth for 24 h at 30 °C were used to prepare the inocula. Cells were collected by centrifugation (8000× *g*, 15 min), washed twice with sterile deionized (DI) water, resuspended in sterile DI water, and then used as inoculum. The fermentation treatment was established with uninoculated juice (UI) as a control. Briefly, only either the single inoculum of *La* or *Td* was inoculated in the JA juice to an initial concentration of approximately 5 log CFU/mL for *La* and *Td* treatment, respectively, while a mixture of both strains at 5 log CFU/mL was inoculated in the JA juice for *La* + *Td* treatment. Uninoculated JA juice was used as a control. A summary of the fermentation process is provided in Table 1. The fermentation process was conducted in 270 mL polypropylene bottles containing 200 mL of JA juice for 24 h at 30 °C. After fermentation, the samples were split into two portions. The first portion was immediately used for microbial enumeration. The second portion was centrifuged at 8000× *g* for 15 min to separate the microbial cells. The resulting supernatant was then frozen at −40 °C for subsequent analysis.

### 2.4. Microbial Enumeration

For the enumeration of viable yeast and LAB counts, the samples were serially diluted in sterile Ringer’s solution (NaCl 1.125 g/L, KCl 0.0525 g/L, CaCl_2_ 0.03 g/L, and NaHCO_3_ 0.025 g/L). The diluted samples were plated in YEPD medium for the yeast count and MRS medium for the LAB count. The plates were incubated at 25 °C for 48 h for the yeast count and at 37 °C for 48 h for the LAB count. Subsequently a single colony of each microbe was enumerated and expressed as Log CFU/mL of the juices.

### 2.5. Physicochemical Analysis

#### 2.5.1. Color Measurement

A color evaluation was performed using a NR200 Precision Colorimeter (Shenzhen Threenh Technology Co., Ltd., Shenzhen, China), and values of L*, a*, b*, H*, and c* were obtained. The L*, a*, b*, H*, and C* values indicate lightness, greenness (negative) to redness (positive), blueness (negative) to yellowness (positive), hue, and chroma, respectively. The total color difference (ΔE) was calculated using the following equation:

(1) $$\text{∆}E={\left[{\left(L_{0}-L^{*}\right)}^{2}+{\left(a_{0}-a^{*}\right)}^{2}+{\left(b_{0}-b^{*}\right)}^{2}\right]}^{0.5}$$

where *L*₀, *a*₀, and *b*₀ represent the initial color values at the start of fermentation, and L*, a*, and b* represent the color values of the samples being tested.

#### 2.5.2. Determinations of pH, Total Titratable Acidity, and Total Soluble Solids

The pH of all JA juice samples was measured using a digital pH meter (Consort NV, Turnhout, Belgium). To determine the total titratable acidity (TA), 10 mL of each sample was diluted in 50 mL of deionized water and the then titrated with 0.1 M NaOH solution using phenolphthalein as an indicator. Titratable acidity was then calculated and expressed as percent lactic acid using the following equation:

TA (%) = (mL of 0.1 M NaOH) × 0.09(2)

The total soluble solid (TSS) content of the juice samples was determined with a refractometer (Atago Model N-1a, Fukaya, Japan).

#### 2.5.3. Free Alpha Amino Acid Content Analysis

Free alpha amino nitrogen (FAN) content was determined using the EBC-ninhydrin method [26]. In brief, 2.0 mL of the sample and 1.0 mL of the ninhydrin reagent were combined in a test tube. The mixture was then heated for 16 min in a constantly boiling water bath, cooled to 20 °C, and 5.0 mL of the diluted solution was then added. The absorbance of the mixture was measured at 570 nm. Glycine (0.14 mM) was used as the standard, and the results are expressed as glycine equivalent in accordance with the following Equation (3):


(3) $$\mathrm{FAN}(\mathrm{mg}/\mathrm{mL})=\frac{{\left(A_{570}\right)}_{\mathrm{sample}}}{{\left(A_{570}\right)}_{\mathrm{glycine}\;\mathrm{standard}}}\times 2$$

#### 2.5.4. Total Phenolic Content Analysis

Total polyphenol content was determined by the Folin–Ciocalteu reagent method modified from Zhong et al. [27]. In brief, 0.5 mL of the JA juice sample was placed in a test tube containing 0.25 mL of 2 M Folin–Ciocalteu reagent and vortexed (Vortex genie 2, Scientific Industries, New York, NY, USA). The mixture was combined with 3.75 mL of DI water and 0.5 mL of 20% (*w*/*v*) sodium carbonate. It was then left in a dark place for 2 h. The absorbance of the final mixture was measured at 750 nm using a spectrophotometer (T80 UV, PG Instruments, Leicestershire, UK). Gallic acid was used as a standard, and the results are expressed as milligrams of gallic acid equivalents (GAEs) per mL of sample.

#### 2.5.5. Antioxidant Activity Assay

The ABTS (2,2′-azino-bis(3-ethylbenzthiazoline-6-sulfonic acid) radical scavenging capacity of the JA juice samples was evaluated using the method described by Wongputtisin et al. [28], with slight modifications. In this method, a 14 mM ABTS solution was mixed with a 5 mM potassium persulfate solution and allowed to react at room temperature in the dark for 12–16 h. The resulting radical cation solution was then diluted to an appropriate concentration before being used. For the assay, 50 µL of the JA juice sample was combined with 2.5 mL of the ABTS radical solution. After a 3 min reaction time, the absorbance of the mixture was measured at 734 nm using the following equation:

(4) $$\mathrm{ABTS}\;\mathrm{scavenging}\;\mathrm{capacity}\;(\%)=\left[\frac{{A_{734}\;\mathrm{at}\;3\;\min-A}_{734}\;\mathrm{at}\;0\;\min}{A_{734}\;\mathrm{at}\;0\;\min}\right]\times 100$$

### 2.6. Antimicrobial Activity Assay

Inhibitory and bactericidal concentrations of JA juice samples were tested in four bacteria cultures comprised of *B. cereus* TISTR 747, *Escherichia coli* TISTR 527, *Staphylococcus aureus* TISTR 746, and *Salmonella* Typhimurium TISTR 1472 using the two-fold dilution technique. This was performed with slight modifications that were adopted according to those employed in previous studies [29]. In brief, nutrient broth containing an inoculum of 10^6^ CFU/mL and serial concentrations of the JA juice samples (0.03–0.99 mL/mL) were evaluated. Minimal inhibitory concentration (MIC) values were taken as representative of the lowest JA juice concentration that prevented visible bacterial growth after 24 h of incubation at 30 °C and the minimum bactericidal concentration (MBC) as representative of the lowest concentration that completely inhibited bacterial growth.

### 2.7. Metabolomic Analysis

The fermented samples (100 μL) were placed in microtubes and resuspended with prechilled 80% (*v*/*v*) methanol through vortexing. The samples were incubated on ice for 5 min and centrifuged at 15,000× *g*, 4 °C for 20 min. The supernatant was diluted to a final concentration containing 53% (*v*/*v*) methanol using LC-MS grade water. The resultant samples were transferred to HPLC vials and subject to LC-MS/MS analysis. The UHPLC-MS system consisted of the Dionex Ultimate 3000 UHPLC system coupled with an Orbitrap Q Exactive Focus mass spectrometer (Thermo Scientific, Waltham, MA, USA). The samples were injected with an autosampler system kept at 4 °C in a 10 μL injection. Liquid separation was achieved using a C18 column (Poroshell 120 EC-C18, 2.7 μm × 100 mm, Agilent, Santa Clara, CA, USA). Gradient chromatography was carried out with 0.1% formic acid as Solvent A and acetonitrile with 0.1% (*v*/*v*) formic acid as Solvent B. The flow rate was 0.25 mL/min. Run time was 45 min with the following gradients: 5% B from 0.0–5.0 min, 5–95% B from 5.0–25.0 min, 95–100% B from 25.0–26.0 min, 100% B from 26.0–35.0 min, 100–5% B from 35.0–36.0 min, and 5% B until 45.0 min to re-equilibrate the column. Positive electrospray ionization (ESI) was utilized. Data acquisition was achieved in the full scan/ddMS2 mode. The full scan resolution was set at 35,000 with an 85–1000 *m*/*z* scan range. The discovery ddMS2 was set at a 17,500 resolution with a 3.0 isolation window, while the collision energy (NCE) was 15. The HESI-II working parameters were as follows: spray voltage at 3 kV, capillary temperature at 350 °C, sheath gas flow rate at 30 psi, auxiliary gas flow rate at 10 au, and auxiliary gas heater temperature at 300 °C. Experimental MS data in the mzXML format were used for metabolite identification. Metabolite peaks were identified and validated using MAVEN2 (metabolomics analysis and visualization engine) [30,31] with a mass error of ± 10 ppm. The metabolites identified with MAVEN2 were validated manually based on peak characteristics. The validated data were used for statistical analysis performed on the MetaboAnalyst 6.0 platform available at https://www.metaboanalyst.ca/MetaboAnalyst/ (accessed on 6 July 2024).

### 2.8. Anti-Inflammatory Assay

The in vitro anti-inflammatory activity of the compounds was investigated using the method described by Ameena et al. [32], with slight modifications. Different concentrations of JA juice samples ranging from 0.625 to 10 mg/mL were analyzed for anti-inflammatory activity. In brief, the reaction mixture contained a total volume of 5 mL, consisting of 0.2 mL of egg albumin, 2.8 mL of phosphate-buffered saline, and 2 mL of samples. It was incubated at 70 °C for 5 min. Subsequently, the absorbance was measured at 660 nm after cooling. Inflammatory inhibitory activity (%) was calculated using the following equation:

(5) $$\%\;\mathrm{Inhibition}=\left[\frac{{\left(A_{660}\right)}_{\mathrm{sample}}}{{\left(A_{660}\right)}_{\mathrm{control}}-1}\right]\times 100$$

The results are expressed as IC_50_ (concentration providing 50% of inhibition of albumin thermal denaturation).

### 2.9. Anticancer Activity Assay

JA juice samples, in concentrations ranging from 0.1 to 1000 µg/mL, were tested for cytotoxic activity in HT-29 colon cancer cells using the SRB (sulforhodamine B) assay [33]. Briefly, the cells were plated at a density of 1.0 × 10^4^ cells/well in 96-well plates. The cells were then exposed to 5 serial concentrations of the samples (0.1–1000 µg/mL) for 24 h. After incubation, the cells were fixed with a 50% (*w*/*v*) trichloroacetic acid solution, incubated at 4 °C for 1 h and stained with 50 µL of 0.4% SRB solution for 30 min at room temperature (25 ± 2 °C). The absorbance was measured at 540 nm. Cisplatin was used as the standard anticancer drug. The results are expressed as IC_50_ values and represent the concentrations needed for the inhibition of 50% cell growth.

### 2.10. Statistical Analysis

The results are expressed as the mean ± standard deviation (SD) values of three repetitions. Analysis of variance (ANOVA) was used to analyze the relationship between samples. Duncan’s new multiple range test (DNMRT) was used to analyze pairwise comparisons of the mean values when there was a significant difference (*p* < 0.05).

## 3. Results and Discussion

Fruit juices are a crucial source of bioactive compounds including phenolics, flavonoids, vitamins, and minerals. However, to ensure food safety, prevent spoilage, and extend shelf life, the proper processing of fruit juices is essential. Traditional fruit juice processing typically involves thermal pasteurization and relies on the natural acidity of the juice. While effective for safety and preservation, these processes can alter the composition of juice and reduce the bioavailability of its nutrients, potentially diminishing the benefits to the consumer. Therefore, fresh JA juice was not included in this study.

### 3.1. Microbiological Properties of Fermented JA Juices

**Figure 1:**
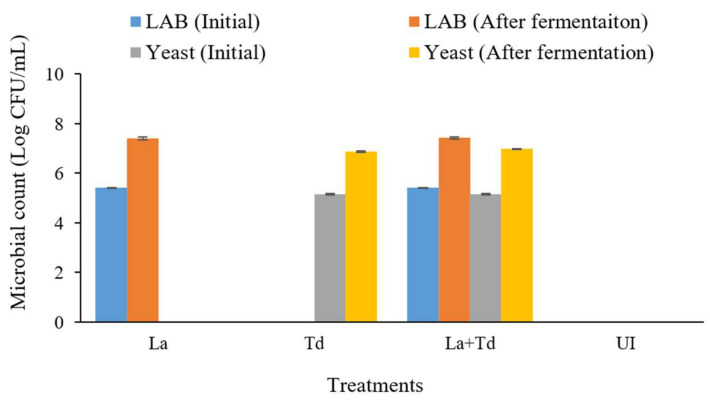
Viable cell growth in JA juices fermented using different inoculation protocols; JA juices inoculated with *La*, *L. acidophilus*; *Td*, *T. delbrueckii*; *La* + *Td*, *L. acidophilus* + *T. delbrueckii*.

LAB are generally considered safe for consumption due to their long history of use, with certain species recognized as probiotics that provide health benefits. While many LAB species are harmless to humans, not all can be classified as “generally recognized as safe” (GRAS) from a regulatory perspective [23,34]. *La*, in particular, is one of the most important LAB in the food industry and holds a GRAS status [34]. This has led to numerous efforts aimed at enhancing their survival in food products, as LAB are crucial for maintaining the desired quality of these foods. *Torulaspora delbrueckii* has been considered for use in improving the aroma profiles in beverages [35,36]. This study revealed that JA juice promoted the growth of *La* and *Td* by 7.4 and 7.0 log CFU/mL, respectively, after 24 h of fermentation (Figure 1). The viable counts of LAB and yeast in the *La*-inoculated and *Td*-inoculated JA juice samples increased after 24 h of fermentation to 2 and 1.7 log CFU/mL, respectively. In comparison, increases of 2.0 and 1.8 log CFU/mL, respectively, were observed in the *La* + *Td*-inoculated JA juice samples. Certain strains of LAB exhibited antagonistic effects against foodborne pathogens, including bacteria, yeast, and filamentous fungi [37]. Conversely, certain strains of yeast, including *Td*, were reported to exhibit inhibitory activity against LAB [38,39]. Therefore, a mixed culture of yeast and bacteria could affect microbial growth in various manners [40,41]. However, during the co-culture fermentation of JA juice, no antimicrobial activity was observed between *La* and *Td*. Similar findings were reported by Bujna et al. [42], where *La* increased by 2.5 log CFU/mL in apricot (*P. armeniaca*) juice after incubation at 37 °C for 24 h, while only a 0.8 log CFU/mL increase was noted in apple juice fermented with *La* [43]. Apricot juice has been found to serve as an effective matrix for LAB growth due to its rich content of nutrients, such as carbohydrates, vitamins, and minerals [42,44]. To meet the recommended probiotic requirements for health benefits, viable cell counts should range between 6 and 11 log CFU/mL, depending upon the strain and the product [45,46]. Most LAB grow more slowly under low pH conditions and can lose viability due to acid damage [47]. However, despite the low pH of the initial JA juice, the results indicate that *La* can still grow and produce metabolites by utilizing various compounds in the juice as nutrient sources.

### 3.2. Physicochemical Properties of Fermented JA Juices

The effects of *La* and *Td* on the physicochemical properties of fermented and unfermented JA juice samples are presented in Table 2. Color is one of the most important food parameters, as it can significantly influence consumer acceptability [48]. In this study, all color parameters significantly changed after fermentation (*p* < 0.05). The lightness (L*) parameter decreased, while the redness (a*), yellowness (b*), chroma (C*), hue angle (H*), and total color difference (∆E*) parameters increased after fermentation. Total color difference is commonly used as a color quality parameter as it can quantify the magnitude of color differences between treatments and control samples after 24 h of incubation. In this case, it was employed to quantify the magnitude of color in the JA juices before fermentation. According to Wibowo et al. [49], perceivable color differences can be analytically classified as not noticeable (0–0.5), slightly noticeable (0.5–1.5), noticeable (1.5–3.0), well visible (3.0–6.0), and significant (>6.0). After fermentation, the ∆E* values were 0.06, 3.29, 2.99, and 2.18 in the uninoculated, *La*, *Td*, and *La* + *Td* inoculated JA juices, respectively. The most distinct color difference, classified as well visible, occurred when the juice was fermented with *La*. The color differences in the JA juices fermented with *Td* and a mixed culture of *La* and *Td* fell into the noticeable classification, while the uninoculated JA juice exhibited no perceptible change. This result indicates that the color differences in the fermented JA juice can be visually distinguished.

**Figure 2:**
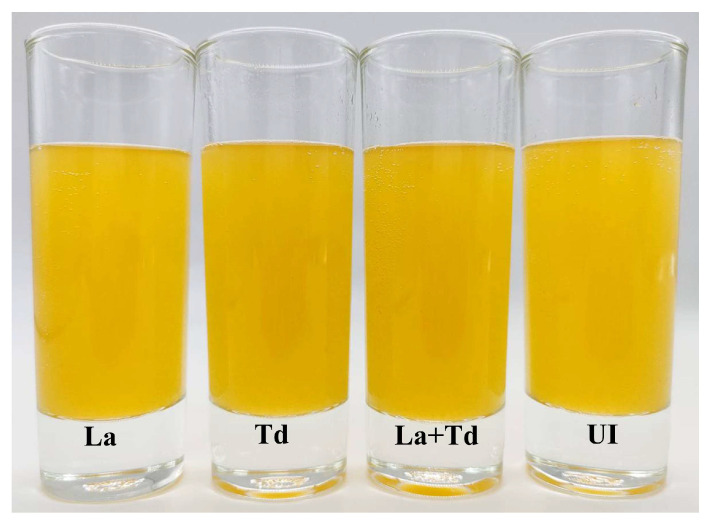
JA juices from different inoculation protocols: UI, uninoculated JA juice and JA juices inoculated with *La*, *L. acidophilus*; *Td*, *T. delbrueckii*; *La* + *Td*, *L. acidophilus* + *T. delbrueckii*.

In addition to the differences in color, chroma (C*) and hue (H*) provided further insights into the color characteristics of the JA juice samples. Before fermentation, C* and H* values were 3.00 and 33.27, respectively. These values increased after fermentation, indicating an increase in color intensity and a shift toward yellowish region. This suggests that fermentation can enhance yellowness. The hue angle values, which ranged from 33.27 to 43.58, confirm that the color of all JA juices appear orange (Figure 2), which is consistent with our visual observations. The rise in b* values, indicating a stronger yellowish tone, further corroborates the observed color change. These results align with the findings of Cele et al. [50], who noted that b* values were significantly affected by inoculation protocols.

Acidity, pH, and total soluble solids (TSSs) are the key indicators in fermented products [45] that are crucial for assessing the sensory quality of beverages and can significantly influence consumer perception [51]. The pH values of all JA juices were nearly identical, while a slight increase in titratable acidity (TA) was observed in the inoculated JA juices (0.60–0.63%) when compared with the uninoculated JA juice (0.58%). A co-culture of *La* and *Td* did not affect the TA and TSS parameters during JA juice fermentation. The consistent pH value observed in this study could be attributed to the buffer capacity of the JA juice matrix [52], while the higher acidity in the *La*-inoculated JA juice could have resulted from the presence of organic acids, primarily lactic acid, produced during lactic acid fermentation through the consumption of sugars [53]. According to Sayın et al. [54], yeast can produce organic acids, which explains the increase in TA observed in the *Td*-inoculated JA juice. Previous studies have also reported on the ability of *Td* to produce organic acids. Accordingly, Bressani et al. [55] described an increase in citric acid and succinic acid during the fermentation of coffee and navel oranges.

TSSs largely determine sweetness and are related to the sugar content of fruit juices. Significant differences in sugar consumption were observed among the three inoculation protocols during the fermentation of JA juices (*p* < 0.05). The *Td*-inoculated juice and *La* + *Td*-inoculated juice exhibited slightly lower TSS values when compared with the La-inoculated juice. This result suggests that *Td* may have a higher sugar demand for growth than *La* during JA juice fermentation.

A sufficient supply of nitrogen and diverse nitrogen sources are critical for the growth and fermentation activity of LAB and yeast, significantly affecting both metabolite production and sensory quality [56,57]. LAB require complex organic nitrogen sources, such as amino acids and peptides [58]. *Saccharomyces* yeasts favor nitrogen sources, like ammonia, glutamine, and asparagine [59], while non-*Saccharomyces* yeasts display distinct amino acid consumption profiles [60]. These profiles are influenced by various factors including the type of nitrogen source [59], microbial strain, sugar content, fermentation conditions [61], inoculation protocol [57], and the ratio of free amino acids [62]. The free amino nitrogen (FAN) levels, which represent nitrogen compounds that yeast can assimilate or metabolize during fermentation [63], vary significantly across different inoculation protocols in JA juice fermentation. JA juice inoculated with *La* + *Td* had the lowest FAN content (Table 2), showing an 85% decrease due to fermentation. In contrast, JA juices inoculated with *La*- and *Td*-inocula showed 16% and 69% decreases in FAN, respectively. These findings underscore the influence of microbial species and inoculation protocols on nitrogen consumption during JA juice fermentation.

Extensive scientific research has shown that LAB fermentation not only prolongs the shelf-life of plant-based products, but also enhances their antioxidant capacity and total phenolic content [64,65,66]. In this study, no significant differences in antioxidant activity were observed between inoculated and uninoculated JA juice. However, fermenting JA juice with *La*, either alone or in a co-culture, led to a 7–10% increase in total phenolic content when compared to fermentation with *Td* and the uninoculated JA juice. While phenolic compounds are major contributors to antioxidant activity, they do not fully account for it, as vitamin C and carotenoids also play a role [67]. In addition, fermentation conditions, such as substrate composition, temperature, time, and LAB strains, can influence LAB activity, leading to differences in antioxidant capacity and total phenolic content [64,65]. Consequently, the impact of *La* on increasing the antioxidant capacity of JA juice was not determined.

### 3.3. Antimicrobial Properties of Fermented JA Juices

The inhibitory effect observed in this study could be attributed to the antimicrobial properties of the fermented JA juice, LAB, and yeast. The antimicrobial effects of LAB are associated with the production of various active metabolites during fermentation, including organic acids, hydrogen peroxide, diacetyl, carbon dioxide, fatty acids, and bacteriocins [37,66]. In this context, LAB exhibit varying degrees of antagonistic activity against pathogens through three primary mechanisms: the production of organic acids that create an acidic environment, competition for nutrients (depleting essential resources), and the formation of antimicrobial compounds [23]. Many strains of yeast, including *Td*, also possess the ability to secrete inhibitory metabolites [68,69]. The ability of UI-JA juice, as well as the inoculated JA samples obtained after fermentation to inhibit the growth of four foodborne pathogenic bacteria at MIC and MBC levels across a range of JA sample dilutions (31–990 µg/mL), is presented in Table 3. The MICs varied notably depending upon the specific samples and bacterial species. For *La*- and *Td*-inoculated JA juices, the MICs were in the ranges of 124–248 µL/mL and 248–495 µL/mL, respectively, while for the UI-JA juice, MICs were observed between 248 and 49.5 µL/mL. The MBC values were consistently equal to or higher than the MIC values across all tested species. Differences in MBC levels were observed between strains, with recorded MBC values ranging from 248 to 990 µL/mL, while no inhibitory activity was noted for the tested samples. In contrast, the fermented JA juice produced by co-culture inoculation exhibited the highest MIC level, reaching 990 µL/mL for all tested bacteria. This surpassed the results of the other three inoculation protocols. Additionally, the co-culture inoculation showed no inhibitory activity in the MBC test. Notably, the reduced inhibitory activity of the co-culture inoculation observed in this study could have resulted from the complexity of the fermented matrix and the intricate microbial metabolic dynamics [70].

### 3.4. Metabolite Profile of Fermented JA Juices

**Figure 3:**
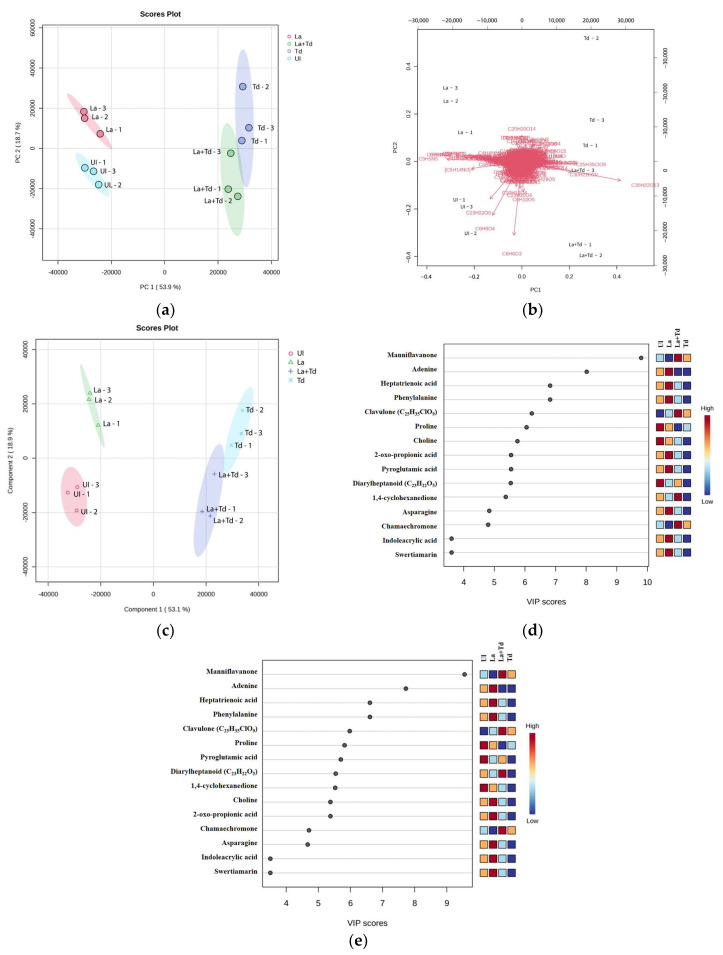
Principal component analysis (PCA) score plot (**a**), biplot (**b**), PLS-DA score plot (**c**), and PLS-DA VIP results of the metabolites (**d**,**e**) established in JA juices fermented using different inoculation protocols.

To more comprehensively analyze the metabolite differences in the four JA juice samples, unsupervised principal component analysis (PCA) and supervised partial least-squares discriminant analysis (PLS-DA) methods were employed to conduct a multivariate statistical analysis of the LC-MS/MS-acquired metabolomes, which likely contributed to the overall quality of the JA juice. A total of 995 metabolites was identified in the four experimental groups. Based on PCA analysis, two principal components (PCs) accounted for 72.50% of the overall variance (Figure 3). PC1 explained 53.8% of the total variance and influenced the differences between the UI-JA juice and *La*-inoculated JA juice samples obtained from *Td*- and *La* + *Td*-inoculated JA juices (Figure 3a). Additionally, PC2 accounted for 18.7% of the total variance, separating UI-JA juice and *La* + *Td*-inoculated JA juice from *La*- and *Td*-inoculated JA juices.

Overall, the PCA results indicate that the juice samples’ metabolite compositions during fermentation are distinguishable based on the inoculation protocols. Moreover, the PCA loading plot and biplot (Figure 3b) suggest that manniflavanone is the primary metabolite attributed to the JA juices obtained from single or co-culture inoculation protocols with *Td*, while diarylheptanoid (2′-6′-Dihydroxy-4′-methoxy-3′-(2-hydroxybenzyl) dihydrochalcone) and 1,4-cyclohexanedione were the predominant contributors to the UI-JA juice. The impact of microbial strains and inoculation techniques on metabolite profiles was consistent with the findings reported in several previous studies [20,36,71].

PLS-DA effectively eliminated noise and identified the most relevant metabolites for distinguishing between groups [72]. According to the PLS-DA score plot, the *La*-inoculated JA samples were completely separated from the other fermentation samples (*Td*- and *La* + *Td*-inoculated), while the *Td*- and *La* + *Td*-inoculated samples had less of an overlap when compared with the PCA results. This observation indicated that the main metabolites in the *La*-inoculated samples differed from those in the *Td*- and *La* + *Td*-inoculated samples, which shared similar main flavor characteristics. Metabolite discriminant variables contributing to the PLS-DA model were ranked along with the variable importance in the projection (VIP) scores. Based on the VIP scores (≥1.0), the higher the VIP scores of the metabolites, the more significant their contribution to the differences between groups [20]. The top 15 metabolites that strongly contributed to the variations included manniflavanone, adenine, heptatrienoic acid, phenylalanine, clavulone, proline, choline, 2-oxo-propionic acid, pyroglutamic acid, diarylheptanoid, 1,4-cyclohexanedione, asparagine, chamaechromone, indoleacrylic acid, and swertiamarin (Figure 3d,e). These results correlate with the PCA biplot, which indicates that manniflavanone serves as a significant discriminant between two clusters: JA juice samples with and without *Td*- inoculation.

Several studies have shown that certain bioactive compounds, such as polyphenols, can interact with polysaccharides in plant cell walls, namely cellulose, hemicellulose, and pectin, via hydrogen bonding, hydrophobic interactions, adsorption, and pi–pi interactions [73]. These interactions suggest that compounds obtained from plant materials may be released through enzymatic hydrolysis by certain enzymes, such as proteases, esterases, glycosidases, and pectinases, which are produced by non-*Saccharomyces* yeasts, like *Torulaspora* [20]. The manniflavanone detected in this study may be a result of such enzymatic reactions. This suggests that *Td* is closely associated with manniflavanone liberation during JA juice fermentation. Further metabolite characterization and pathway/enrichment analysis will help elucidate the *Td* metabolic pathways that are specifically involved in JA juice fermentation.

### 3.5. Anti-Inflammatory Properties of Fermented JA Juices

Anti-inflammatory action is a key component of the health benefits associated with fruits and vegetables [74]. In addition, numerous studies have demonstrated that fermentation with LAB can enhance the anti-inflammatory properties of these fruits and vegetables [75,76]. JA, in particular, is known for its health-promoting effects including its anti-inflammatory benefits [77,78]. Kim et al. [17] reported that JA juice fermented with a mixed culture of LAB, specifically *Lpb. plantarum* and *Lbs. casei*, could serve as a natural therapeutic agent for colitis and intestinal inflammation.

According to the dominant metabolic profile of co-culture fermentation, the fermented JA juice obtained from *La* + *Td* inoculation was selected to assess the anti-inflammatory activity when compared with UI-JA juice. The results indicate that the unfermented JA juice has an IC_50_ value of 2.16 mg/mL against inflammation, whereas the fermented JA juice with the *La* + *Td* co-culture inoculation exhibits no anti-inflammatory activity (Table 4). Considering the complexity of inflammatory mechanisms, further studies using different analysis techniques are required to better understand the anti-inflammatory properties of JA juices fermented with mixed LAB and yeast cultures [79].

Furthermore, the absence of anti-inflammatory activity from this study might be due to the degradation of active compounds by enzymes produced by microbial strains or other biochemical reactions during fermentation [80,81]. These findings are in agreement with the understanding that selecting appropriate raw materials, microbial strains, and inoculation strategies is crucial for potentially enhancing the functional properties of fermented JA juices [62].

### 3.6. Anticancer Properties of Fermented JA Juices

The bioactive compounds present in fruits and vegetables have exhibited anticancer activity, which is recognized as a positive health effect [82]. Considering that colorectal cancer is the third most common cancer worldwide [83], the anticancer potential of JA juice samples on HT-29 cell lines was examined in this study. Previous studies have reported on the potential anticancer effects of JA [19,84], and the results of this study provide further evidence that fermented JA, using a mixed culture of LAB and non-*Saccharomyces* yeast, also possesses functional anticancer properties. Some microbial metabolites also exhibit anticancer properties; therefore, both *La* + *Td*-fermented and UI-JA juices might exhibit comparable anticancer activity against HT-29 cells. However, *La* + *Td*- and UI-JA juices showed IC_50_ values of 823.37 and 754.87 µg/mL, respectively (Table 5). Although, these values are significantly lower than those of the reference compound (Cisplatin), these juices indicate a significant potential in contributing to the prevention or adjunct treatment of colorectal cancer [83]. This study highlights the promising health benefits of both fermented and unfermented JA juices.

## 4. Conclusions

Fermented beverages have a promising future, enhanced by the rising demand for functional drinks among health-conscious consumers. Additionally, the growing interest in plant-based functional beverages is driving this demand. Although much research is still needed, fermented JA appears to hold significant potential in this exciting sector. This study provides an overview of the hypothesis that various fermented JA juices produced using LAB (*La*) and non-*Saccharomyces* yeast (*Td*) can serve as functional beverages. These juices offer potential health benefits to consumers. The juice fermented with *La* demonstrated superior antimicrobial properties. However, the juice fermented using a co-culture of *La* and *Td* improved the particular metabolic profiles, making it a promising candidate as a functional fermented beverage. This study provides strong evidence that both single and co-culture fermentation processes are effective techniques that affect a product’s characteristics. The presence of viable LAB in the fermented juice would likely be recognized as an alternative probiotic carrier to consumers. Future research should focus on the process optimization of fermented JA juice production and evaluate other health functionalities. From a food industry perspective, the findings obtained from this study can serve as guidance for enhancing product sales and provide significant advantages for the commercial development of functional food products.

## Figures and Tables

**Table 1:** Fermentation treatment for Japanese apricot juice fermentation.

Strains	Fermentation Treatment			
	*La*	*Td*	*La* + *Td*	UI
*L. acidophilus*	+	−	+	−
*T. delbrueckii*	−	+	+	−

**Table 2:** Physicochemical properties of JA juices fermented by different inoculation protocols.

Properties	*La*	*Td*	*La* + *Td*	UI
L*	26.58 ± 0.01 ^d^	27.08 ± 0.16 ^c^	27.91 ± 0.02 ^b^	29.09 ± 0.02 ^a^
a*	3.82 ± 0.01 ^a^	3.64 ± 0.09 ^b^	3.38 ± 0.01 ^c^	2.50 ± 0.00 ^d^
b*	3.29 ± 0.02 ^a^	3.34 ± 0.02 ^a^	3.21 ± 0.00 ^b^	1.66 ± 0.06 ^c^
∆E	3.29 ± 0.02 ^a^	2.99 ± 0.07 ^b^	2.18 ± 0.03 ^c^	0.06 ± 0.03 ^d^
C*	5.04 ± 0.02 ^a^	4.94 ± 0.08 ^b^	4.66 ± 0.00 ^c^	3.00 ± 0.04 ^d^
H*	40.73 ± 0.22 ^b^	42.57 ± 0.62 ^a^	43.58 ± 0.19 ^a^	33.64 ± 0.96 ^c^
pH	2.99 ± 0.01 ^a^	2.99 ± 0.01 ^a^	2.99 ± 0.01 ^a^	2.98 ± 0.00 ^b^
Total acidity (% as citric acid)	0.60 ± 0.00 ^b^	0.63 ± 0.01 ^a^	0.62 ± 0.01 ^ab^	0.58 ± 0.00 ^c^
Total soluble solid content (°Brix)	13.00 ± 0.00 ^a^	12.20 ± 0.00 ^b^	12.16 ± 0.05 ^b^	13.03 ± 0.05 ^a^
Free alpha amino nitrogen content (mg/L)	6.31 ± 0.28 ^b^	2.34 ± 0.17 ^c^	1.14 ± 0.28 ^d^	7.53 ± 0.19 ^a^
Antioxidant activity^ns^ (% ABTS scavenging)	56.14 ± 2.69	54.74 ± 2.52	53.86 ± 2.36	52.22 ± 2.53
Total phenolic content (mg/L)	273.33 ± 13.57 ^a^	244.00 ± 3.46 ^b^	267.00 ± 8.88 ^a^	249.33 ± 1.15 ^b^

Mean values ± standard deviations followed by different letters within a row indicate significant differences using Duncan’s multiple range test at *p* < 0.05; ns denotes mean values that are not significantly different (*p* > 0.05); UI, uninoculated JA juice and JA juices inoculated with *La*, *L. acidophilus*; *Td*, *T. delbrueckii*; *La* + *Td*, *L. acidophilus* + *T. delbrueckii.*

**Table 3:** Minimum inhibitory concentrations (MICs; µL/mL) and minimum bactericidal concentrations (MBCs; µL/mL) of JA juices fermented using different inoculation protocols.

Treatment	*B. cereus*		*E. coli*		*S. aureus*		*S. typhimurium*	
	MIC	MBC	MIC	MBC	MIC	MBC	MIC	MBC
*La*	124	248	248	−	248	495	248	990
*Td*	248	248	248	990	495	990	495	990
*La* + *Td*	990	−	990	−	990	990	990	−
UI	248	248	495	990	495	990	495	−

Note: −, no inhibitory activity was observed at the tested maximum concentration of 990 µL/mL; UI, uninoculated JA juice and JA juices inoculated with *La*, *L. acidophilus*; *Td*, *T. delbrueckii*; *La* + *Td*, *L. acidophilus* + *T. delbrueckii.*

**Table 4:** Anti-inflammatory properties of co-culture fermented and UI-JA juices.

Sample	Anti-Inflammatory Activity
	IC_50_ (mg/mL)
*La* + *Td*-inoculated JA juice	NA
UI-JA juice	2.16 ± 0.10
Diclofenac diethylammonium (Standard anti-inflammatory drug)	0.29 ± 0.02

NA denotes no activity.

**Table 5:** Effects of fermented co-culture and UI-JA juices on anticancer activity against the HT-29 colorectal cancer cell line.

Sample	Anticancer Activity
	IC_50_ (µg/mL)
*La* + *Td*-inoculated JA juice	823.37 ± 218.60
UI-JA juice	754.87 ± 202.76
Cisplatin (standard anticancer drug)	0.985 ± 0.00

## Data Availability

The original contributions presented in the study are included in the article. Further inquiries can be directed to the corresponding authors.
